# Near-Infrared Excited Mn^4+^- and Nd^3+^-Doped Y_2_SiO_5_ Luminescent Material with Flower-like Morphology for Plant-Centric Lighting Applications

**DOI:** 10.3390/molecules30214161

**Published:** 2025-10-22

**Authors:** Liza Rani Deka, Marta Michalska-Domańska, Shubhra Mishra, D. S. Kshatri, M. C. Rao, Neeraj Verma, Vikas Dubey

**Affiliations:** 1 Department of Physics, North-Eastern Hill University, Shillong 793022, Meghalaya, India; lizarani.kamrup@gmail.com; 2 Institute of Optoelectronics, Military University of Technology, 00-908 Warsaw, Poland; 3 Department of Physics, Government Naveen College Gudhiyari, Raipur 492009, Chhattisgarh, India; 4 Department of Physics, Shri Shankarachraya Institute of Professional Management and Technology, Raipur 492001, Chhattisgarh, India; 5 Department of Physics, Andhra Loyola College, Vijayawada 520008, Andhra Pradesh, India; raomc72@gmail.com; 6 Department of Physics, Govt. G.S.G.PG. College, Balod 491226, C.G., India

**Keywords:** controlled-environment agriculture, phosphor, deep-red emission, LED, enhanced plant growth

## Abstract

Confronted with increasing global food demands, diminishing arable land, and climate volatility, controlled-environment agriculture with advanced red and far-red LED lighting can enhance photosynthesis and optimize plant growth. This investigation reports the generation of a Mn^4+^/Nd^3+^ co-doped Y_2_SiO_5_ phosphor with a Nd^3+^ concentration ranging from 0.1 to 2.5 mol% via a solid-state synthesis method, aiming to enhance red and far-red emission for plant cultivation LEDs. For the Y_2_SiO_5_:Mn^4+^ (1 mol%), Nd^3+^ (2 mol%) phosphor, the phase integrity, nanostructured morphology, elemental mapping, and vibrational characteristics were examined using XRD, Rietveld analysis, FTIR, SEM, and EDX. Nd^3+^ ions act as near-infrared excitation mediators, ensuring efficient Nd^3+^ → Mn^4+^ energy transfer upon 808 nm excitation, and this leads to pronounced red photoluminescence from Mn^4+^ ions that covers the range of 640–710 nm, exhibiting strong emission peaks centered at 650nm, 663nm, and 685nm, coinciding with the absorption band of phytochromes and chlorophyll. The optimal emission intensity was accomplished for a Nd^3+^ doping concentration of 2 mol%, beyond which concentration quenching occurred. The material produced a strong, concentrated deep red emission with CIE coordinates near (0.73, 0.27) and a high color purity of 98.96%, making it well-suited for photosynthetic activation. A phosphor-integrated red pc-LED was fabricated, and Tulsi plants were grown under this LED during the winter in Meghalaya, a period critical for plant growth due to the low ambient light. Over a 30-day period, the plants exhibited enhanced height and leaf development, demonstrating the practical potential of Mn^4+^/Nd^3+^ co-doped Y_2_SiO_5_ for energy-efficient, wavelength-optimized horticultural lighting.

## 1. Introduction

Food is a fundamental requirement for life and is universally provided by plants. Plants are the ultimate sources of nutrients, highlighting their pivotal role in sustaining life on Earth. Four main pigments found in plants are chlorophyll A, chlorophyll B, phytochromes P_r_ and P_fr_. When plants absorb light at specific wavelengths, particularly blue, red, and far-red, it triggers pigment synthesis. This process stimulates and regulates plant growth and development, making light crucial for plant growth [1,2,3,4]. Blue light (400–500 nm), red light (600–700 nm), and far-red light (700–780 nm) directly influence phototropism, photosynthesis, and light morphology, respectively. In contrast, yellow-green light has been observed to be less effective for supporting plant growth [5,6].

Among these pigments, the photoreceptor proteins phytochromes P_r_ and P_fr_ are the most significant, as reported in many studies [7,8,9,10,11,12,13,14]. The two categories of phytochromes are inactive P_r_ and active P_fr_. Pr is stimulated by red light (660 nm), while Pfr is activated by far-red light (730 nm). Upon absorbing red light, the phytochrome molecule changes from the P_r_ to the P_fr_ form. When it absorbs far-red light, it shifts back to the P_r_ form. Exposure to far-red light (730 nm) causes the plant to accumulate the P_r_ form. This accumulation signals the presence of taller plants nearby that block direct sunlight. As a result, the plant increases its growth effort to overcome this obstacle. This is why far-red light promotes plant growth. Plant chlorophyll and carotenoids are also sensitive to blue and red light and thus play a pivotal role in plant growth [15]. Red light (610–700 nm) boosts vegetative flowering, budding, and internodal elongation. Far-red light (~720 nm) also helps foster plant growth and supports photosynthesis [16]. The wavelengths mentioned above are directly absorbed to enhance growth, while photosynthetic bacteria, commonly found in nature, absorb near-infrared (NIR) light. These bacteria, often found in nature, facilitate biological nitrogen fixation in plant roots and convert inorganic nitrogen salts, indirectly promoting growth [17,18]. In conclusion, red, far-red, and NIR light enrichment support robust plant development by enhancing photosynthetic activity.

Traditionally, cultivation has been performed in open fields for ages, which has made the plants and crops vulnerable to environmental adversities such as floods, droughts, unwanted rains, and cyclones, hindering plant growth and crop quality due to insufficient sunlight, which the plants need. To overcome these challenges, modern agriculture, particularly indoor plant growth, is an effective approach in which plants are cultivated in a controlled environment of light, air, water, and temperature [19,20,21,22,23,24,25,26]. Indoor farming utilizes artificial lighting sources, such as fluorescent, metal halide, high-pressure sodium, and incandescent bulbs, which emit broad, non-adjustable spectra. This results in inefficient energy utilization due to partial energy dissipation and leads to suboptimal plant growth [27,28,29,30]. The integration of light-emitting diodes (LEDs) in contemporary agriculture signifies a notable advancement in addressing challenges as a supplementary light source. Recent progress has emerged, highlighting their efficacy in high brightness, luminosity, minimal thermal output, and adjustable light spectrum [31,32]. Phosphor-converted light-emitting diodes (pc-LEDs) are the preferred choice of LED, enabled by advanced manufacturing techniques that integrate these systems with various single-color emitting phosphors, activated by near-ultraviolet (n-UV) or blue semiconductor diodes to produce light. Control over the spectral profile can be achieved by altering the phosphor composition [31,33]. In this context, phosphors emit light via a widespread photophysical phenomenon known as photo-luminescence, which relies on the crystal structure of the host lattice, as well as the activator ion and the sensitizer introduced to enhance the luminescence intensity [5,34,35,36].

Inorganic phosphors exhibit various crystal structures, such as perovskites, garnets, oxides, sulfides, and nitrides, with prospects for future advancements [34]. However, silicates have attracted considerable interest in recent years owing to their chemical and thermal stability, as well as their high luminescent intensity. The diverse structures of silicates provide unique coordination settings for activator ions, rendering them a promising luminescent medium and yielding emissions spanning the n-UV, blue, green, orange, red, NIR, and white spectra, as discussed in many research studies [37,38,39,40]. More precisely, yttrium silicates are favored due to their prevalent monoclinic structure, which accommodates luminescent centers and enhances charge transfer, rendering them exceptional photo-luminescent materials [41]. Extensive studies in various literatures indicate that they are predominantly doped with rare earth ions, including Y_2_SiO_5_:Eu^3+^ [42], Y_2_SiO_5_:Ce^3+^ [43], Y_2_SiO_5_:Tb^3+^ [44], and Y_2_SiO_5_:Sm^3+^ [45], among others.

In recent years, transition metals, particularly Mn^4+^, have been extensively explored alongside rare earth ions due to their cost-effectiveness, ease of preparation, and tunable emission spectra in the far-red region [46]. The Mn^4+^ ions possess incompletely filled orbitals with an electronic configuration of 3d^3^, resulting in excitation spectra that exhibit allowed transitions of ^4^A_2g_ → ^4^T_2g_ and ^4^A_2g_ → ^4^T_1g_, as well as a spin-allowed transition of ^4^A_2g_ → ^4^T_1g_. The emission spectra consist of the ^2^E_g_ → ^4^A_2g_ transition, thereby emitting red or far-red light, highlighting its potential for optical applications in plant growth [47,48]. Numerous research works have been undertaken on Mn^4+^ ions, such as Sr_2_GdGaO_5_:Mn^4+^ [46], SrLaMgTa_1-y_Al_y_O_6_:Mn^4+^ [49], and Ba_2_LaSbO_6_:Mn^4+^ [50], which emit the requisite red or far-red light, rendering Mn^4+^ ions an excellent and favored choice of activator. The rare earth Nd^3+^ ions are of the favored sensitizers in our study due to their energy levels being organized in a stepwise or ladder-like structure, which enables absorption across a broad spectral range from UV to NIR [51]. Neodymium ions (Nd^3+^) exhibit several unique absorption peaks, with prominent peaks detected at around 730, 808, and 865 nm, corresponding to transitions from the ^4^F_7/2_, ^4^F_5/2_, and ^4^F_3/2_ excited states to the ^4^I_9/2_ ground level [52,53]. The characteristics of Nd^3+^ have been extensively studied by Yujin Chen and his team in relation to the La_2_(WO_4_)_3_:Nd^3+^ phosphor [51]. Devarajulu et al. [54] examined Yb^3+^/Nd^3+^-codoped silicate-based oxyfluoride glasses, offering valuable insights into the sensitization properties of Nd^3+^.

Acknowledging the crucial relevance of red and far-red light for plant growth, this research focuses on the production of phosphor materials that emit light efficiently in these wavelength areas to promote and foster plant growth. This study focuses on the design and investigation of phosphors co-doped with Mn^4+^ and Nd^3+^ ions using an appropriate Y_2_SiO_5_ host to meet the required specifications. Within this framework, the Mn^4+^ transition metal ion serves as the primary luminescent center, emitting in the red and far-red spectral regions, whilst the Nd^3+^ rare earth ion functions as the sensitizer, facilitating broad-spectrum energy absorption and transfer. The transition metal has been employed as an activator ion within the prospective host Y_2_SiO_5_, including a promising direction for future investigation. This study examines the luminescent properties and elucidates the energy transfer mechanisms between Nd^3+^ and Mn^4+^, facilitating the assessment of the material’s potential application in developing next-generation LED-based artificial lighting systems for indoor horticulture. This research aims to promote energy conversion and photomorphogenic responses in plants through the development of energy-efficient, spectrum-customizable light sources, representing a significant advancement in sustainable agricultural operations. Therefore, the effective design and optimization of Y_2_SiO_5_:Mn^4+^/Nd^3+^ phosphor demonstrates the capability for advancing plant growth LEDs that utilize up-conversion photoluminescence to enhance agricultural efficiency.

## 2. Results and Discussion

### 2.1. XRD Analysis

**Figure 1:**
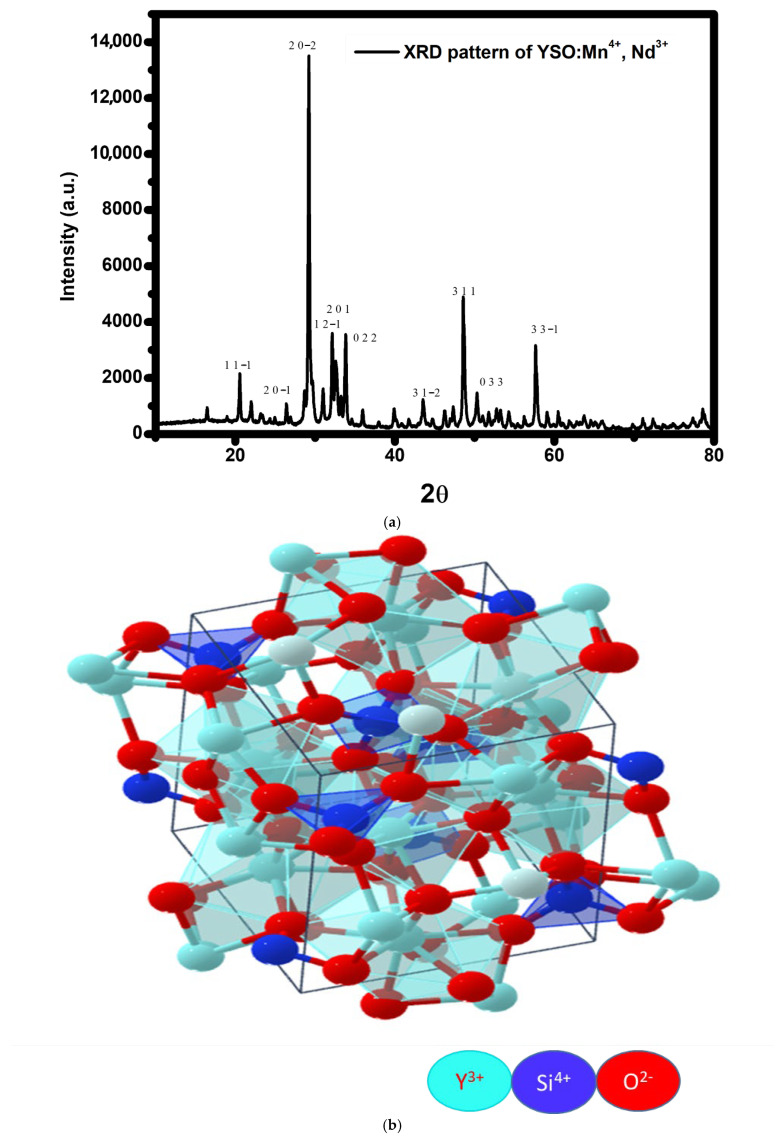

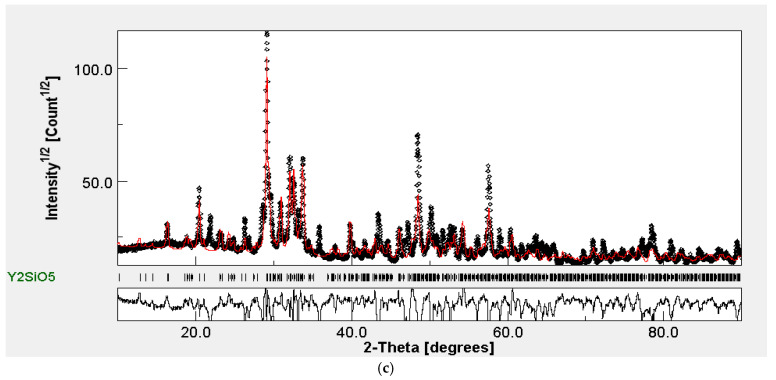
(**a**)XRD pattern of Mn^4+^ (1 mol%) and Nd^3+^ (2 mol%)-doped Y_2_SiO_5_ phosphor. (**b**) Crystal structure of Y_2_SiO_5_ phosphor. (**c**) Refinement pattern of XRD data.

To evaluate the phase purity and structural integrity of the synthesized Y_2_SiO_5_, Mn^4+^ (1 mol%), Nd^3+^ (2 mol%) phosphor, and powder X-ray diffraction (XRD) measurements were carried out at room temperature. The XRD pattern of the as-synthesized phosphor, depicted in Figure 1a, closely aligns with the conventional monoclinic Y_2_SiO_5_ structure (Figure 1b) characterized by *P*2_1_/*c* space group, as documented in the Materials Project database (MP-554420) with cell parameters a = 6.68 Å, b = 6.85 Å, and c = 9.04 Å. In this structure, two crystallographic sites (Y_1_ and Y_2_) are filled by Y^3+^ ions, with each yttrium site separately coordinated by adjacent oxygen atoms. Doping introduces the substitution of Nd^3+^ ions for Y^3+^ sites due to their similar ionic radii (r), valency, and coordination number (CN) (r[Nd^3+^] = 1.109 Å, CN = 8 compared to r[Y^3+^] = 1.075 Å, CN = 8), leading to little lattice distortion. Similarly, Mn^4+^ ions (r[Mn^4+^] = 0.530 Å, CN = 6) are anticipated to nominally substitute Si^4+^ ions (r[Si^4+^] =0.40 Å, CN=4) in the Y_2_SiO_5_ host lattice due to their equivalent charge state [55]. However, the significant disparities in ionic radii and coordination environments between Mn^4+^ and Si^4+^ suggest that direct substitution may be constrained. Consequently, Mn^4+^ ions are expected to occupy octahedral interstitial or defect sites or to form charge-compensated defect complexes to uphold overall charge neutrality and structural stability, as supported by analogous findings in similar oxide hosts [56,57]. This dual inclusion mechanism explains the observed phase purity and stable crystal structure despite the lattice mismatch. The co-doped sample exhibits no secondary or impurity phases, confirming its phase purity. Utilizing the conventional *P*2_1_/*c* monoclinic structure, the principal diffraction patterns were indexed, and the corresponding (hkl) planes were assigned accordingly. Prominent reflections were observed at 2θ ≈ 29.22564°, 48.62457°, and 57.69076°, corresponding to the (hkl) planes (20-2), (311), and (331), respectively. This alignment with the standard diffraction data further validates the phase uniformity and structural stability of the material. The average crystallite size (D) of the synthesized phosphor was estimated using Scherer’s formula:

(1) $$D\;=\;\frac{k\lambda }{\beta \cos\theta }$$

where D is the crystallite size, k is the shape factor (0.9), λ is the X-ray wavelength (1.54056 Å for Cu Kα radiation), β is the FWHM of the chosen diffraction peak (in radians), and θ is the corresponding diffraction angle. The average crystallite size of the material calculated using Scherer’s formula is found to be 29.52 nm (determined from Table 1) using the 2θ values and the FWHM (β) of the most intense peaks corresponding to the respective planes (hkl), which indicates the nanocrystalline nature of the sample. Table 1 represents the hkl values of the 10 peaks together with their 2θ values, FWHM values, and crystallite sizes.

Figure 1c illustrates the Rietveld refining of the XRD pattern of the produced phosphor. Rietveld refinement was performed using the MAUD software (version 2.99) tool, employing structural constraints derived from the standard data MP-554420 from the Materials Project. There are a few faint, low-intensity peaks that do not correspond to typical patterns, potentially resulting from minor structural abnormalities, localized lattice defects, or instrumental effects. Despite these effects, the primary diffraction peaks remained closely matched with the reference pattern, and no distinct peaks indicating impurity phases were detected, confirming that the dopants are effectively integrated into the host lattice without compromising its overall structural integrity.

### 2.2. FTIR Study of Mn^4+^ and Nd^3+^Co-Doped Y_2_SiO_5_

**Figure 2:**
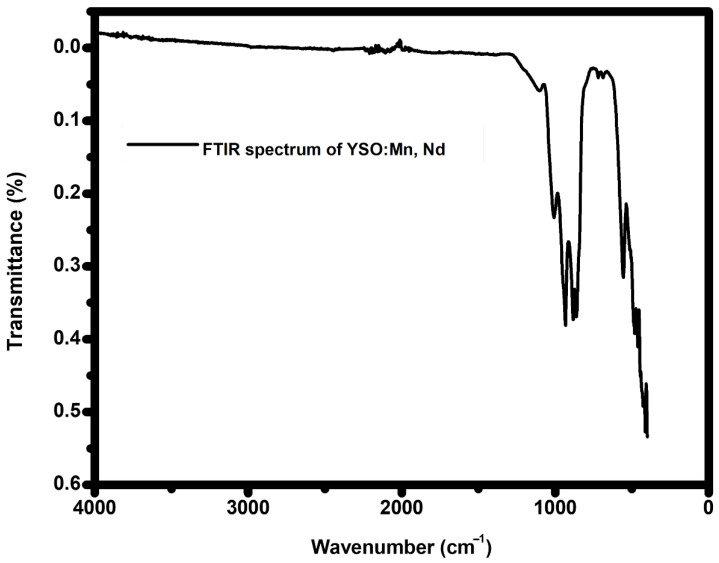
FTIR spectrum of Mn^4+^ (1 mol%), Nd^3+^ (2 mol%)-doped Y_2_SiO_5_ phosphor.

Fourier Transform Infrared (FTIR) spectroscopy was employed to ascertain the vibrational characteristics of the host lattice and to assess the structural modification induced by Mn^4+^ and Nd^3+^ co-doping in the Y_2_SiO_5_ matrix. The FTIR spectrumfor the phosphor Y_2_SiO_5_:Mn^4+^ (1 mol%), Nd^3+^ (2 mol%) was observed in the range of 350–4500 cm^−1^, as shown in Figure 2. The broad range of 1500–4000 cm^−1^ exhibits minimal vibration, showing the lack of significant OH^−^ (hydroxyl) or organic-related vibrational features, thus confirming the exceptionally high purity of phosphor, which can be ascribed to the extreme temperatures utilized during the synthesis procedure [58,59]. Notable vibrational bands are detected at 1102, 1008, 932, 883, 719, 686, and 556 cm^−1^. The prominent peaks at 1102 cm^−1^ and 1008 cm^−1^, corresponding to the asymmetric stretching vibrations of Si-O-Si bonds, indicate the structural integrity of the silicate network [60,61]. The infrared peak at 937 cm^−1^ corroborates the existence of the fundamental Si–O stretching vibration [62]. The absorption feature at 883 cm^−1^ is attributed to the Si–O stretching motion within the SiO_4_ tetrahedral framework, while the absorption peak at 686 cm^−1^ results from the asymmetric stretching of Si–O linkages. The peaks identified at 556 and 719 cm^−1^ correspond to the stretching or bending vibrations of Y–O bonds in the host structure [63]. The peak at 556 cm^−1^ is also associated with symmetric Mn-O-Mn vibration, indicating the incorporation of Mn^4+^ into octahedral coordination [64], as well as Nd-O stretching [65]. Table 2 enumerates all the vibrations along with their respective peaks. These vibrational signals demonstrate the compositional purity and robust stability of the synthesized phosphor system, demonstrating the effective incorporation of dopant ions into the host lattice without inducing structural damage.

### 2.3. FEGSEM Study

**Figure 3:**
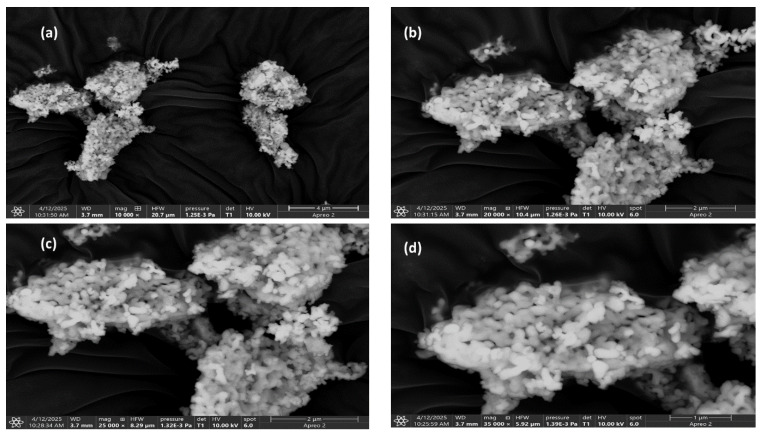
(**a**–**d**) FEGSEM images of Mn^4+^ (1 mol %), Nd^3+^ (2 mol %)-doped Y_2_SiO_5_ phosphor.

FEGSEM (field emission gun scanning electron microscopy) is conducted to obtain a clearer understanding of phosphor Y_2_SiO_5_:Mn^4+^ (1 mol%), Nd^3+^ (2 mol%) by examining its morphology and microstructural characteristics at four distinct magnifications, as illustrated in Figure 3a–d, which showcases striking FEGSEM images.

Figure 3a, captured at a magnification of ×10,000, distinctly illustrates the formation of substantial agglomerated clusters of particles within the 4 µm range of the prepared phosphor, indicating their origin from the solid-state reaction. These non-uniform clusters, characterized by robust intergranular fusion and porous structure, enhance light scattering and absorption, hence promoting optical activities.

Figure 3b presents an FEGSEM image at ×20,000 magnification, revealing a distribution within the 2 µm range. This allows for an in-depth examination of the rough surface, which illustrates the partial merging of grains. This phenomenon can be attributed to dopant-enhanced sintering, as well as the effective incorporation of impurities, specifically Mn^4+^ and Nd^3+^, within the carrier matrix Y_2_SiO_5_. The interparticle bridges facilitate smoother phonon-mediated energy transfer between luminescent centers, which aids in maintaining consistent emission intensity, positioning our material as a promising phosphor for LED applications.

Figure 3c presents an image at magnification of ×25,000, showcasing a tightly woven microcrystalline structure characterized by flower-like or flake-like particle assemblies, which emphasize a well-defined crystalline network. The fine-grained morphology improves surface uniformity while decreasing the likelihood of non-radiative losses. The densely packed grains promote efficient energy transfer, resulting in a high surface-to-volume ratio that creates additional active sites for phonon interaction, ultimately leading to enhanced photonic emission.

Figure 3d illustrates a highly porous and organized agglomeration at a magnification of 35,000 with a distribution in the 1 µm range. The image depicts the granular surface created, and the observed porosity exhibits scattering characteristics that subsequently improve photon–lattice interaction. The presence of pores serves as an energy barrier that inhibits non-radiative recombination, thereby prolonging the excited-state lifetimes of luminescent centers.

The SEM analysis indicates that the synthesized phosphor exhibits promising structural features, including a large surface area, porosity, and compact microcrystals, which enhance luminescent efficiency. Importantly, the observed microstructural flower-like morphology underscores the material’s suitability as a potential LED phosphor.

### 2.4. EDXS Analysis

**Figure 4:**
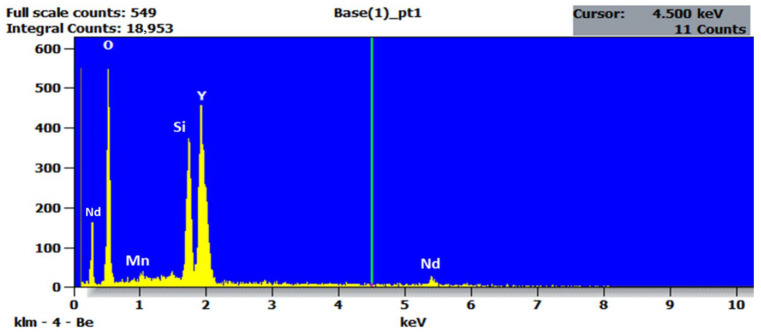
EDXS spectrum of Mn^4+^ (1 mol%), Nd^3+^ (2 mol%)-doped Y_2_SiO_5_ phosphor.

Energy Dispersive X-Ray Spectroscopy (EDXS) is employed to confirm the elemental composition and validate the successful incorporation of dopants into the host matrix of our synthesized sample, YSO:Mn^4+^ (1 mol%), Nd^3+^ (2 mol%), prepared using the solid-state synthesis method. The EDX spectrum was acquired from the same area examined in SEM imaging to achieve composition-informed visualization of the microstructure, as illustrated in Figure 4. The spectrum clearly displays peaks associated with yttrium, silicate, oxygen, manganese, and neodymium, in accordance with the expected stoichiometry of Y_2_SiO_5_:Mn^4+^, Nd^3+^ (1.5%). The significant presence of dopants Mn^4+^ and Nd^3+^ indicates effective incorporation into the host matrix. The absence of foreign elemental peaks indicates the high purity of the synthesized sample. The accurate identification and measurements of trace-level dopants, specifically Mn^4+^ and Nd^3+^ at a low atomic concentration, underscore the sensitivity of the EDXS approach, which is crucial for optimizing the optical properties of luminescent phosphors. The elemental composition presented in both atomic percentage (at %) and weight percentage (wt %) is summarized in Table 3. The EDXS analysis confirms that the synthesized sample Y_2_SiO_5_:Mn^4+^, Nd^3+^ possesses the intended elemental composition, establishing a robust basis for subsequent optical and functional characterization.

### 2.5. PL Emission and PLE Study of Y_2_SiO_5_:Mn^4+^ (1 Mol%), Nd^3+^ and Energy Transfer Mechanism

The Mn^4+^ ions are a widely utilized class of activators due to the incompletely filled orbital with a 3d^3^ electric configuration, which subsequently gives rise to sharp emission in the red and far-red region ranging from 600 to 720 nm due to the spin-forbidden ^2^E_g_ → ^4^A_2g_ transition [47,48,49,50,64]. The emission well overlaps with the phytochrome pigments, but the confined emission bandwidth of Mn^4+^ intrinsically restricts its spectral distribution, therefore reducing its applicability in plant cultivation lighting, where preference has shifted toward a broader luminescence profile matching chlorophyll-sensitive wavelengths [66]. Nevertheless, this limitation can be addressed by the incorporation of Nd^3+^ as a co-dopant, as it introduces spectral broadening and the formation of supplementary emission bands through efficient energy transfer and dual ion emission [67]. This co-doping strategy increases the emission bandwidth and enhances excitation energy utilization through an energy transfer mechanism, which makes the phosphor more appropriate for uses like LED-based horticulture lighting applications that call for broad and effective red to NIR emission. Based on the findings, the synthesized phosphor Y_2_SiO_5_:Mn^4+^, Nd^3+^ demonstrates up-conversion luminescence, enhancing its usability additionally by turning lower-energy photons into higher-energy emissions.

**Figure 5:**
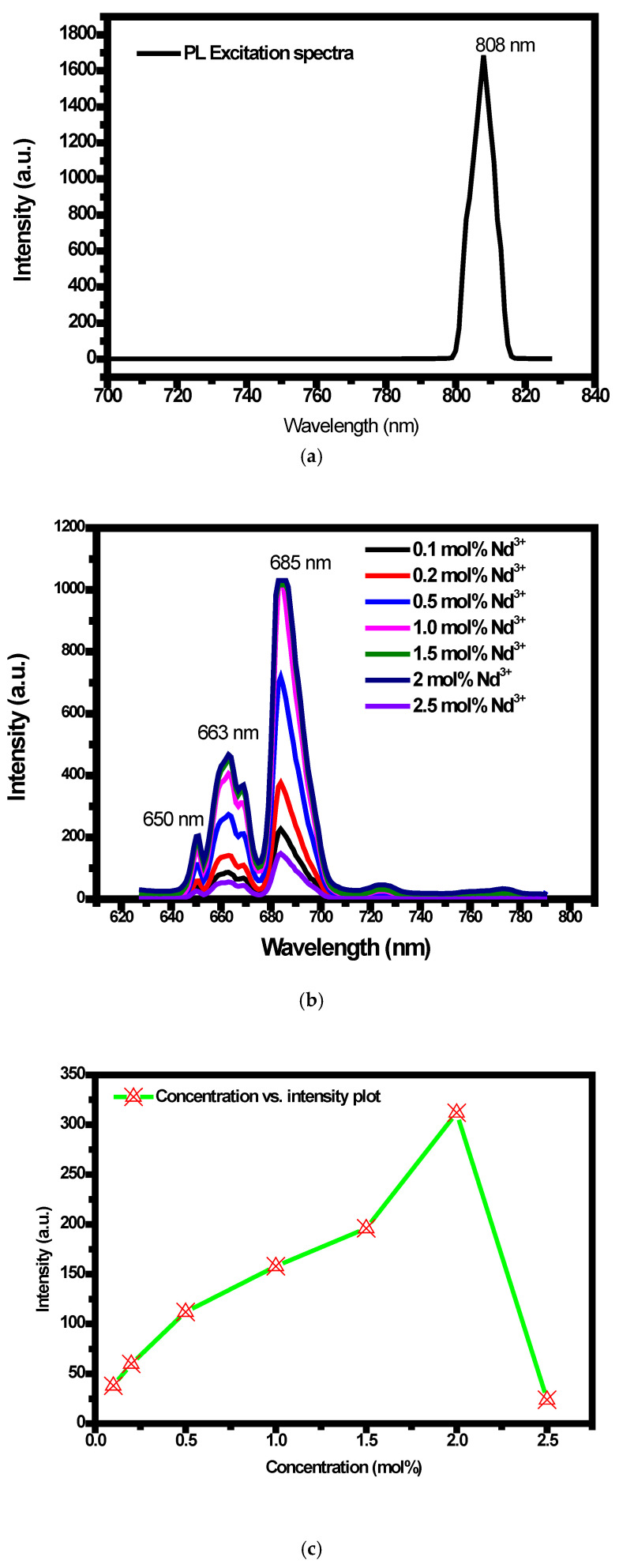

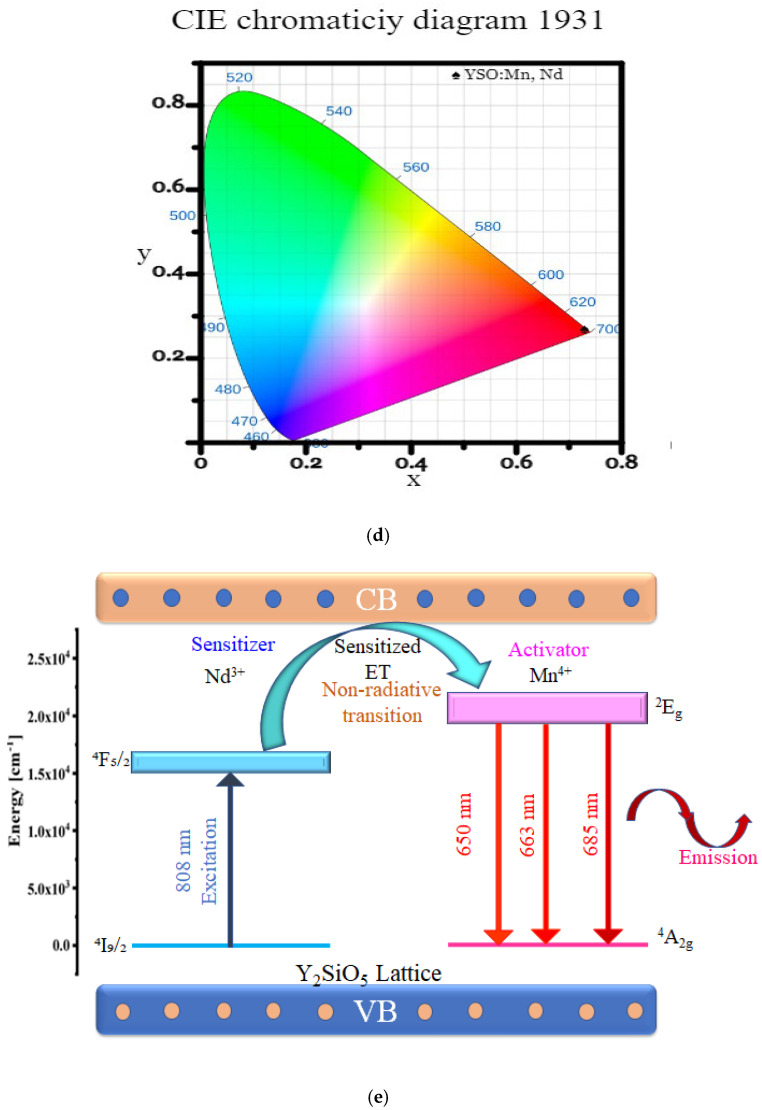
(**a**) PL excitation spectrum of Y_2_SiO_5_:Mn^4+^ (1 mol%), Nd^3+^ (2 mol%) phosphor. (**b**) PL emission spectra of Y_2_SiO_5_:Mn^4+^, Nd^3+^ phosphor with a constant concentration of Mn^4+^ and varying concentration of Nd^3+^ (0.1–2.5 mol%). (**c**) Concentration vs. intensity of PL emission spectrum. (**d**) CIE 1931 coordinate of Mn^4+^ (1 mol%), Nd^3+^ (2 mol%)-doped Y_2_SiO_5_ phosphor (X = 0.73, Y = 0.27). (**e**) Energy level diagram for the charge transfer phenomenon.

Systematic investigation has been carried out on the synthesized Y_2_SiO_5_:Mn^4+^ (1 mol%), Nd^3+^ (0.1 mol%, 0.2 mol%, 0.5 mol%, 1 mol%, 1.5 mol%, 2 mol%, and 2.5 mol%) phosphor to assess the optical characteristics and to confirm the energy transfer between the Nd^3+^ and Mn^4+^. The observations and measurements were executed under normal temperature conditions. The interionic energy transfer dynamics between Nd^3+^ and Mn^4+^ inside the Y_2_SiO_5_ host lattice were examined through a comprehensive analysis of the PL and PLE spectra. As shown in Figure 5a, the PLE spectrum of Y_2_SiO_5_:Mn^4+^, Nd^3+^ exhibits a prominent excitation band centered at ~808nm when monitoring Mn^4+^ emissions near 650nm. Spectroscopically, this band is linked to the ^4^I_9_/_2_ → ^4^F_5_/_2_ excitation transition of Nd^3+^ [52,53]; this clearly indicates that Nd^3+^ ions can be excited efficiently in that wavelength region. The excitation features reflect the fact that energy transfer occurs from Nd^3+^ to Mn^4+^, leading to the excitation of Mn^4+^ indirectly when radiated with 808 nm.

The photoluminescence spectra of Y_2_SiO_5_:Mn^4+^, Nd^3+^ phosphors across different Nd^3+^ concentrations (0.1–2.5 mol%) to optimize the emission intensity were recorded as shown in Figure 5b, resulting in emission from 640 to 710 nm under the excitation around 808 nm attributed to the spin-forbidden ^2^E_g_ → ^4^A_2g_ transitions of Mn^4+^ ions [67]. The presence of distinct emission peaks around 650 nm and 663 nm and 685 nm, attributed to the ^2^E_g_ → ^4^A_2g_ transitions of Mn^4+^ ions, provides red emission. Though Mn^4+^ emission is primarily attributed to the ^2^E_g_ → ^4^A_2g_ transition, the observed spectral features comprise multiple peaks due to Stark level splitting, vibronic coupling, and site-related variation. The increase in Nd^3+^ content led to a noticeable, sharp (broadened), and more intense 685 nm band of the emission profile between 680 and 700 nm, ascribed to strengthened interionic energy exchange and concurrent luminescence involving Nd^3+^ [68]. Given this observation, a conclusion can be made that Nd^3+^ → Mn^4+^ energy transfer leads to the conversion of the 808nm NIR light into broadened red emission (640–710 nm), which correlates with plant photoreceptor sensitivity. Using this co-doping approach leads to enhanced excitation efficiency with extended spectral coverage, which collectively positions Y_2_SiO_5_:Mn^4+^, Nd^3+^ phosphors as reliable phosphors that can be utilized in pc-LED horticultural lighting applications.

Moreover, to gain a deeper understanding of the dopant concentration, a graph depicting the relationship between doping concentration and intensity is presented in Figure 5c. Observational evidence demonstrates a gradual increase in emission intensity with a rising concentration of Nd^3+^ from 0.1 mol% to 2 mol%, attributed to the effective energy transfer from Nd^3+^ to Mn^4+^. A higher concentration of Nd^3+^ ions provides more available sites for energy absorption and transfer to the activator [69]. Nevertheless, the PL intensity exhibits a significant decrease at 2.5 mol% of Nd^3+^. The concentration quenching occurs due to increased Nd^3+^ content, which enhances energy hopping among Nd^3+^ ions and disrupts the energy transfer pathway to Mn^4+^. Consequently, the optimal concentration of Nd^3+^ for achieving the most intense Mn^4+^ emission is determined to be 2 mol%, as evidenced by the graph illustrating the variation of integrated PL with respect to Nd^3+^ concentration; surpassing this threshold results in concentration quenching of the sensitizer, thereby constraining the overall luminescence efficiency. Regulating the dopant concentration enables customized red and far-red emission ratios, which are essential for applications in smart lighting and plant photobiology.

Additionally, to evaluate the color performance of the phosphors, the emission spectra were utilized to calculate the Commission Internationale de l’Éclairage (CIE) chromaticity coordinates, as illustrated in Figure 5d. At a concentration of Nd^3+^ at 2 mol%, the emission chromaticity coordinates (X = 0.73, Y = 0.27) distinctly reside within the deep-red spectrum, as denoted by a cross mark and guiding line on the CIE 1931 diagram, highlighting the superior red output of the Y_2_SiO_5_:Mn^4+^ (1 mol%), Nd^3+^ (2 mol%) phosphor sample. Their corresponding location is indicated in Figure 5d by a cross in the deep-red region.

(2) $$\mathrm{Color}\;\mathrm{Purity}=\frac{\sqrt{{\left(x_{s}-x\right)}^{2}+{\left(y_{s}-y\right)}^{2}}}{\sqrt{{\left(x_{d}-x\right)}^{2}+{\left(y_{d}-y\right)}^{2}}}\times 100\%$$

where (x_s_,y_s_) represent the chromaticity coordinates of the sample, (x_d_,y_d_) represent the coordinates of the dominant wavelength on the spectral locus, and (x,y) signify the coordinates of the reference illuminant. The calculation was conducted using this formula, resulting in a color purity of 98.96%. The phosphor demonstrated deep red emission with CIE coordinates of approximately (0.73, 0.27), resulting in a high color purity of ~98%. This confirms the sample’s suitability for agricultural lighting applications, particularly due to the spectral overlap with the absorption bands of chlorophyll a and b, as well as the absorption profile of phytochrome pigments.

The photophysical mechanisms governing the luminescent properties of Y_2_SiO_5_:Mn^4+^, Nd^3+^ were comprehensively illustrated using an energy level diagram (Figure 5e). The energy transmission process between Nd^3+^ and Mn^4+^ within the Y_2_SiO_5_ lattice is schematically illustrated in Figure 5e. The excitation of Nd^3+^ ions at 808 nm leads to non-radiative energy transfer to Mn^4+^ centers, resulting in several emission peaks across the 640–710 nm spectral range, which corresponds to the spin-forbidden ^2^E_g_ → ^4^A_2g_ transition of Mn^4+^. The emission profile exhibits discrete peaks at around 630, 650, and 685nm, owing to Stark sublevel splitting and site-dependent transitions inside the Mn^4+^ ion environment. This diagram provides a clear visual representation of the energy transfer mechanism, facilitating the comprehension of the spectrum characteristics of PL and PLE (Figure 5e).

### 2.6. Real-World Application: Cultivation of Ocimum Tenuiflorum (Tulsi) Using Sunlight and Red LED Illumination

To create an application-oriented benchmark for assessing the efficacy of red-emitting phosphors like Y_2_SiO_5_:Mn^4+^, Nd^3+^ in regulated plant cultivation, a comparative analysis was performed wherein Tulsi plants were cultivated during the winter season in Meghalaya, a crucial phase for plant growth due to diminished ambient light, initially under natural sunlight and subsequently under a commercially available red LED. Tulsi, or Holy basil, scientifically known as *Ocimum tenuiflorum*, was chosen as a test plant because of its notable medical properties and widespread application in traditional Indian medicine (Ayurveda). Tulsi is renowned for its immune-enhancing, anti-inflammatory, and antitussive attributes, making it very efficacious in addressing coughs, colds, and respiratory ailments. Tulsi is widely cultivated for its medicinal benefits and is essential in herbal treatments for respiratory and seasonal ailments.

**Figure 6:**
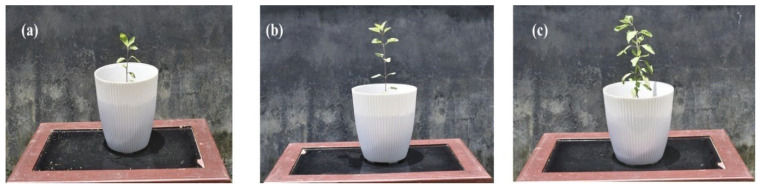
(**a**–**c**) The Tulsi plant was under sunlight within intervals of (**a**) 10 days, (**b**) 20 days, and (**c**) 30 days.

The study was performed in Meghalaya during the winter season, a period typically marked by insufficient sunlight and inadequate growing circumstances due to a predominantly gloomy sky, which is unsuitable for Tulsi cultivation. During this period, a juvenile Tulsi plant 10 cm in height was cultivated in a pot filled with enriched, nutrient-rich soil and exposed to natural sunlight for 30 days during the day and subjected to darkness at night, thus simulating typical outdoor conditions. Growth features were assessed at the three 10-day intervals, as illustrated in Figure 6a–c, indicating growth progression at days 10, 20, and 30 with heights of 13 cm, 15 cm, and 16.5 cm, respectively. The data indicate that the plant has noticeable weak and stunted growth during the period, characterized by little stem elongation and a relatively low rise in leaf count, signifying overall inadequate vegetative development under these light-deficient conditions.

**Figure 7:**
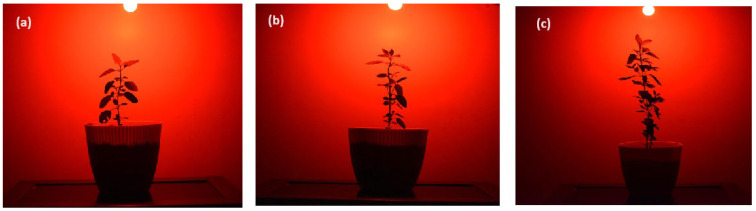
(**a**–**c**) The Tulsi plant was under 16 h of prolonged illumination a day using commercial red pc-LED within intervals of (**a**) 10 days, (**b**) 20 days, and (**c**) 30 days.

To enhance the comprehension of red light’s role in promoting plant growth, a juvenile Tulsi plant of identical beginning height, as utilized in the sunlight experiment, was subsequently grown under red LED light. The experimental investigation was conducted in a controlled atmosphere using commercially available red LED light on Tulsi. The LED emitted deep red light at 1600 lumens per watt, with a wavelength of 680 nm, closely aligning with the emission profile of synthesized phosphor. A juvenile Tulsi plant measuring 10 cm was put in the nutrient-rich soil within a pot and continuously exposed to continuous red illumination from a commercial red-emitting LED for a period of 30 days. The Tulsi plant was subjected to 16 h of continuous light daily, followed by 8 h of darkness. The utilized commercial LED exhibited peak emission in the deep-red spectrum (~680–690 nm), closely matching the luminescence profile of the synthesized Y_2_SiO_5_:Mn^4+^, Nd^3+^ phosphor. This spectral emission notably coincides with the absorption spectra of chlorophyll and phytochrome pigments, hence initiating physiological processes such as photosynthesis and photomorphogenesis [46]. Growth characteristics were monitored at 10-day intervals, illustrated by the visual record in Figure 7a–c. The growth at 10 days, 20 days, and 30 days was 14 cm, 20 cm, and 30cm, respectively. Compared to the plant growth under natural light circumstances, the results revealed a significant enhancement in morphological traits. The Tulsi plant exhibited notable height elongation and a significant increase in leaf count in each observation, as illustrated in the accompanying figures. Additionally, the plant exhibited a pronounced tendency to grow toward the light source, indicating a robust light-induced growth response.

The excellent correlation between the commercial red LED emission profile and the photoluminescence emission of Y_2_SiO_5_:Mn^4+^, Nd^3+^ strongly endorses its prospective use. This experimental configuration served as a dependable model for assessing plants’ responses to deep-red light. These findings further emphasize the horticultural potential of the developed Y_2_SiO_5_:Mn^4+^, Nd^3+^ phosphor as a formidable candidate for solid-state lighting systems, particularly deep red-emitting pc-LEDs, which are intended to enhance plant development. This is particularly significant in regulated environments and low-light conditions, reinforcing the notion that the deep red-emitting Y_2_SiO_5_:Mn^4+^, Nd^3+^ phosphor-based LED can effectively enhance the growth of the Tulsi plant, improving both its morphological and physiological traits.

### 2.7. Plant Growth Application of a Fabricated Y_2_SiO_5_:Mn^4+^, Nd^3+^ Phosphor-LED: Cultivation of Ocimum Tenuiflorum

To assess the feasibility of implementing synthesized Y_2_SiO_5_:Mn^4+^, Nd^3+^phosphor in plant growth-promoting lighting, the phosphor is integrated with an NIR laser, leading to the development of a prototype red-emitting phosphor-converted LED (pc-LED) device. The phosphor powders were synthesized with 1 mol% of Mn^4+^, whereas the concentration of sensitizer Nd^3+^ varied from 0.1 to 2.5 mol%. Research on photoluminescence behavior suggests that an increase in emission intensity was observed at a doping concentration of Nd^3+^, with a maximum at 2 mol%. This increase results from enhanced energy absorption and transfer efficiency between Nd^3+^ and Mn^4+^ to the appropriate level. Consequently, to validate the enhanced red-light emission and LED operational efficacy, a phosphor composition with an optimal concentration of 2 mol% Nd^3+^ is selected for the manufacturing of pc-LEDs. The appropriate phosphor mixture was homogenously blended with a transparent silicone matrix and stimulated using a commercial NIR laser (~808 nm). The phosphor was further subjected to heat curing, resulting in a device with an emission profile of approximately 685 nm with a power output of 1600 lumens per watt. The PL of Y_2_SiO_5_:Mn^4+^, Nd^3+^ demonstrates a pronounced red emission centered at 685 nm, which roughly corresponds to the absorption spectra of phytochrome pigment and chlorophyll. The CIE chromaticity coordinate further validates the deep-red emission feature crucial for indoor horticulture lighting applications. These results affirm that the Y_2_SiO_5_:Mn^4+^, Nd^3+^ phosphor presents a viable alternative to conventional red phosphors in NIR laser-pumped LED setups.

**Figure 8:**
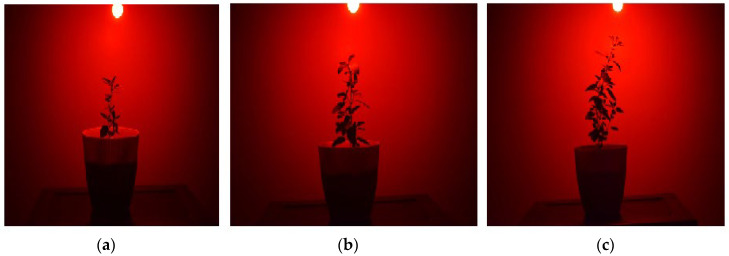
(**a**–**c**) The Tulsi plant was placed under 16 h of prolonged illumination a day using fabricated Y_2_SiO_5_:Mn^4+^, Nd^3+^ phosphor-LED within intervals of (**a**) 10 days, (**b**) 20 days, and (**c**) 30 days.

To enhance comprehension of the capabilities of Y_2_SiO_5_:Mn^4+^, Nd^3+^ phosphors for the production of pc-LEDs designed to facilitate plant development, an experimental study was conducted in a controlled environment on the Tulsi (*Ocimum tenuiflorum*) plant, a species valued for its medicinal and spiritual importance in Hindu culture. A juvenile Tulsi plant was cultivated in nutrient-rich soil within a pot, consistently exposed to constant red illumination from a Y_2_SiO_5_:Mn^4+^, Nd^3+^ phosphor-based pc-LED for a duration of 30 days. The Tulsi plant, measuring 10 cm, was subjected to 16 h of continuous light daily, followed by 8 h of darkness. Growth characteristics were monitored at 10-day intervals, as illustrated in the visual record presented in Figure 8a–c. The plant’s height was measured at 10 days, 20 days, and 30 days with values of 15 cm, 22 cm, and 32cm, respectively. Compared to plants cultivated under natural light conditions, the results demonstrated a significant improvement in morphological traits. The Tulsi plant exhibited notable height elongation and a significant increase in leaf count in each observation, as illustrated in the accompanying figures. Additionally, the plant exhibits a pronounced tendency to grow toward the light source, indicating a robust phototropic response. The findings indicate that the deep red-emitting Y_2_SiO_5_:Mn^4+^, Nd^3+^ phosphor-based LED significantly enhances the growth of the Tulsi plant, improving both morphological and physiological properties. The observations presented here highlight the potential applicability of Y_2_SiO_5_:Mn^4+^, Nd^3+^ phosphor-based deep red pc-LEDs in the advancing field of advanced horticultural techniques and plant growth optimization approaches.

## 3. Experimental Procedure

### 3.1. Preparation of Y_2_SiO_5_:Mn^4+^, Nd^3+^ Phosphors

A series of Y_2_SiO_5_:Mn^4+^ (1 mol%), Nd^3+^ (x mol%) phosphor samples with varying Nd^3+^ doping levels (x = 0.1, 0.2, 0.5, 1, 1.5, 2, and 2.5 mol%) were synthesized through the conventional solid-state reaction technique at elevated temperature. All the suitable precursors—Y_2_O_3_, SiO_2_, MnO_2_, and Nd_2_O_3_—were selected, and they were of analytical grade and employed without additional purification. Based on stoichiometric calculations, the starting materials were proportioned and afterwards manually ground in an agate mortar for 30 min to achieve homogeneity. The homogenized mixture was transferred into an alumina (or corundum) crucible, exposed to calcination at 900 degrees Celsius for an hour, and then sintered at 1250 degrees Celsius for three hours in a muffle furnace under ambient air conditions and boric acid used as a flux [58]. Once naturally cooled to room temperature, the sintered sample was reprocessed by grinding into a fine powder, thereby making it suitable for further characterization.

### 3.2. Characterization

Powder X-ray diffraction (XRD) was conducted on the Y_2_SiO_5_:Mn^4+^ (1 mol%), Nd^3+^ (2 mol%) phosphor sample to assess its crystal structure and phase purity using a Bruker D8 Advance X-ray diffractometer operating in Bragg–Brentano configuration with CuKα radiation (λ = 1.54060 Å). The chemical bonding and functional groups present in the sample were identified and investigated using Fourier Transform Infrared (FTIR) spectroscopy within the spectral range of 400–4000 cm^−1^ using a [Perkin Elmer Spectrum 100 FTIR spectrometer]. The microstructural behavior and particle size of the synthesized phosphor powders were analyzed with a field emission gun scanning electron microscope (FEGSEM), [JEOL JSM-6360]. Elemental distribution and dopant existence were also confirmed via energy-dispersive X-ray spectroscopy (EDX) integrated with an SEM system. Photoluminescence excitation (PLE) and emission (PL) spectra were recorded at room temperature using a fluorescence spectrometer (ocean optics spectrophotometer) in order to study the optical properties of the synthesized samples (Y_2_SiO_5_:Mn^4+^ (1 mol%), Nd^3+^ (0.1 to 2.5 mol%)). To evaluate the color output of the phosphor, the Commission Internationale de l’Éclairage (CIE) chromaticity coordinates, along with the color purity, were calculated from the emission spectra where peak photoluminescence emission is observed.

### 3.3. Tulsi Growth Experiments

The growth of Tulsi (*Ocimum tenuiflorum*), a plant revered in Hindu culture for its medicinal and spiritual significance, was systematically observed. An investigation was carried out during the winter season in Meghalaya, where the same height juvenile Tulsi plant (10 cm) was planted inside pots containing soil with all essential nutrients. The plant was initially illuminated under sunlight in natural lighting conditions. Subsequently, the illumination was switched to a commercially available deep red-emitting LED containing the emission profile that closely aligned with the emission of synthesized Y_2_SiO_5_:Mn^4+^, Nd^3+^ phosphor. The plant was then illuminated using the fabricated red pc-LED based on the synthesized Y_2_SiO_5_:Mn^4+^, Nd^3+^ phosphor.

Plants under LED illumination were irradiated for 16 h per day with an 8 h dark cycle, maintained throughout a 30-day culture period under controlled environmental conditions (temperature of 10–16 °C with standard air circulation). Uniform watering of 50 mL per plant per day was applied across all experiments. Plant growth was monitored at 10-day intervals (i.e., at 10, 20, and 30 days) by recording the increment in plant height and leaf number to analyze the influence of red light on Tulsi growth.

### 3.4. Fabrication of Red-Emitting pc-LEDs Based on Y_2_SiO_5_:Mn^4+^, Nd^3+^ Phosphors

To realize the device-level applicability of the synthesized phosphors, red pc-LEDs were fabricated using the optimized Y_2_SiO_5_:(1 mol%) Mn^4+^, (2 mol%) Nd^3+^ phosphor in plant lighting. The phosphor powder was homogenously mixed with a transparent silicone matrix and excited with a commercial NIR laser (~808 nm). After thermal curing, the devices were packaged and tested using optoelectronic measurement methods. The photoluminescence spectra and CIE colour coordinates were measured in order to assess the red-emission properties. The measured deep-red emission (~685 nm) is consistent with the photosynthetically active spectrum, which indicates the phosphor’s potential in horticultural solid-state lighting.

## 4. Conclusions

The co-doping of Mn^4+^ and Nd^3+^ into the Y_2_SiO_5_ host lattice was successfully achieved via the traditional solid-state method, resulting in a red-light-emitting phosphor with a customized deep-red emission profile and enhanced efficiency. XRD analysis coupled with Rietveld refinement confirmed the phase purity and nanocrystalline nature (~29.5 nm) of the synthesized material. FTIR, SEM, and EDXS confirm the efficient inclusion of elements and the porous, flake/flower-like morphology, which is optimal for enhanced luminescence performance. Photoluminescence studies reveal that Nd^3+^ ions function as NIR sensitizers, facilitating efficient energy transfer Nd^3+^ → Mn^4+^ under 808nm excitation for the ^4^I_9_/_2_ → ^4^F_5_/_2_ transition. The emission spans the area of 640–710 nm, comprising three prominent peaks centered at 650 nm, 663 nm, and 685 nm, which signify deep red emissions associated with the spin-forbidden ^2^E_g_ → ^4^A_2g_ transitions in the Mn^4+^ activator ions. An optimum Nd^3+^ concentration of 2 mol% was determined, beyond which luminescence efficiency declined due to concentration quenching effects. Photometric assessment produced color coordinates (0.73, 0.27) in the CIE 1931 space, exhibiting a high color purity of 98.96%, thereby affirming its correspondence with plant-responsive red spectra crucial to enhance chlorophyll-mediated energy production and phytochrome-mediated morphogenic regulation. To enhance its validation, Tulsi plants were initially grown during the winter season for 30 days in Meghalaya, a crucial growth period. First grown under natural sunlight and then under a commercial LED, with an emission closely resembling the phosphor profile, where the growth under the LED was markedly superior. Subsequently, the synthesized phosphor was effectively included into a constructed red pc-LED, exhibiting emission that closely aligns with the ideal deep-red PL. Tulsi plants cultivated under this LED over the winter season in Meghalaya displayed excellent development, including enhanced height and leaf count, illustrating the practical efficacy of the phosphor-based LED for horticultural lighting. These findings represent a significant advancement in horticultural illumination, demonstrating that deep-red Y_2_SiO_5_:Mn^4+^, Nd^3+^ phosphor-based LEDs can enhance plant development in controlled environments, facilitating energy-efficient and high-performance indoor cultivation systems.

## Figures and Tables

**Table 1:** Crystallite size calculation of prepared phosphor utilizing Scherer’s formula and hkl values.

Peak	B (degree)	B (rad)	2θ	Θ (rad)	Cosθ	0.9λ	B * Cosθ (rad)	D (nm)	hkl
1	0.22538	0.003932	20.5641	0.179365	0.983957	0.13865	0.003869	35.84036	11-1
2	0.19276	0.003363	26.42177	0.230457	0.973562	0.13865	0.003274	42.35292	20-1
3	0.22676	0.003956	29.22564	0.254913	0.967685	0.13865	0.003828	36.22124	20-2
4	0.29306	0.005112	30.99775	0.270369	0.963672	0.13865	0.004927	28.1435	12-1
5	0.98932	0.017258	32.42406	0.28281	0.960275	0.13865	0.016573	8.366263	201
6	0.26124	0.004557	33.83133	0.295084	0.956778	0.13865	0.00436	31.79899	022
7	0.2995	0.005225	43.56326	0.379968	0.928676	0.13865	0.004852	28.57609	31-2
8	0.30354	0.005295	48.62457	0.424114	0.911404	0.13865	0.004826	28.73011	311
9	0.34542	0.006026	50.29503	0.438684	0.905311	0.13865	0.005455	25.41667	033
10	0.30405	0.005304	57.69076	0.503192	0.876048	0.13865	0.004647	29.83947	33-1

**Table 2:** FTIR bands for various modes.

Observed Wavenumber (cm^−1^)	Assigned Vibration	Bond	Ref. No.
1102	Stretching	Si-O-Si	[60]
1008	Stretching	Si-O-Si	[61]
932	Stretching	Si-O	[62]
883	Asymmetric stretching	Si-O	[63]
719	Bending	Y-O	[63]
686	Asymmetric stretching	Si-O	[63]
556	Bending/symmetric/stretching	Y-O/Mn-O-Mn/Nd-O	[63,64,65]

**Table 3:** Quantitative analysis of EDXS.

Element	Molecular Weight (amu)	Weight Percentage (%)	Atomic Percentage (%)
Y	88.906	60.8519	24.625
Si	28.086	9.66055	12.375
O	15.999	27.7933	62.5
Mn	54.938	0.19088	0.125
Nd	144.24	1.50343	0.375

## Data Availability

The data presented in this study are available on request from the corresponding author. The data are not publicly available due to privacy restrictions.
